# Defense Responses and Metabolic Changes Involving Phenylpropanoid Pathway and PR Genes in Squash (*Cucurbita pepo* L.) following *Cucumber mosaic virus* Infection

**DOI:** 10.3390/plants11151908

**Published:** 2022-07-23

**Authors:** Ahmed Abdelkhalek, Lóránt Király, Al-Naji A. Al-Mansori, Hosny A. Younes, Ahmed Zeid, Mohsen Mohamed Elsharkawy, Said I. Behiry

**Affiliations:** 1 Plant Protection and Biomolecular Diagnosis Department, Arid Lands Cultivation Research Institute, City of Scientific Research and Technological Applications, Alexandria 21934, Egypt; 2 Centre for Agricultural Research, Plant Protection Institute, ELKH, 15 Herman Ottó Str., H-1022 Budapest, Hungary; kiraly.lorant@atk.hu; 3 Botany Department, Faculty of Science, Derna University, Derna 417230, Libya; elnajielmansori77@gmail.com; 4 Agricultural Botany Department, Faculty of Agriculture (Saba Basha), Alexandria University, Alexandria 21531, Egypt; hosnyyounes@yahoo.com (H.A.Y.); zeidahmed@alexu.edu.eg (A.Z.); said.behiry@alexu.edu.eg (S.I.B.); 5 Agricultural Botany Department, Faculty of Agriculture, Kafrelsheikh University, Kafr Elsheikh 33516, Egypt; mohsen.abdelrahman@agr.kfs.edu.eg

**Keywords:** plant virus, squash, *Cucumber mosaic virus*, gene expression, GC–MS, HPLC

## Abstract

The current study focuses on the effects of *Cucumber mosaic virus* (CMV) infection on phytochemical changes and pathogenesis- and phenylpropanoid pathway-associated gene activities in squash (*Cucurbita pepo* L.) plants during a time course of 2 to 12 days post inoculation (dpi). The identity of the CMV isolate was confirmed by DAS-ELISA, TEM, and coat protein gene sequence. The CMV infection initially boosts and then suppresses transcript levels of the defense-related genes *PR-1, PR-2, PAL, HQT*, and *CHS* during the investigated time course compared to controls. The expression profile during the time-course study indicated that early, transient induction of *PR-1* occurs during CMV infection, while CMV induced the expression of *PR-2* in systemically infected squash tissues at all time points and suppressed the expression of *PAL* and *HQT* at 8-12 dpi. *CHS* transcript levels fluctuated between up- and down-regulation, but by 12 dpi, *CHS* expression reached its peak. The HPLC and GC–MS analyses of CMV-infected squash extracts revealed that different phenolic, flavonoid, and fatty acid compounds could be induced or suppressed upon CMV infection. In particular, CMV could suppress the synthesis of most phenolic compounds, specifically chlorogenic acid, possibly leading to the virus’s rapid spread.

## 1. Introduction

*Cucumber mosaic virus* (CMV) belongs to the genus *Cucumovirus*, within the *Bromoviridae* family [1,2]. It is one of the most common and economically significant plant viruses globally, with a very diverse host range that includes over a thousand plant species from a hundred families [3,4]. Due to CMV infections, squash suffers from severe foliar mosaic symptoms and fruit deformations. The latter symptom, in particular, has a negative impact on demand in the market [5].

Polyphenols, flavonoids, anthocyanins, phenolic acids, and phenolic terpenes are all produced by plants to regulate critical physiological mechanisms to protect themselves from biotic stress conditions like pathogen oxidative stress [6]. Plants accumulate phenols at the inoculation site to delay the development of invading microbes and limit their spread in plant tissues by increasing the production of reactive species and free radicals [7]. Viral infection of plants causes changes in polyphenolic contents, primarily involving the up- and down-regulation of phenolic compound concentrations as well as antioxidative enzymes such as polyphenol oxidase, superoxide dismutase, and catalase. These alterations cause cell damage, reactive oxidative species (ROS) production, and the activation of pathogen-mediated defense mechanisms, such as the accumulation of e.g., salicylic acid [8]. In the pumpkin, *Yellow vein mosaic virus* (YVMV) infection raised the levels of phenolic constituents, which boosted the plant’s immune system; these phenolic constituents were increased by 73% in infected plant leaves and by 300% in infected fruits as compared to healthy controls [9,10]. CMV infection raised the levels of phenols in tomatoes, leading to increased cell wall lignification and thus playing a key role in plant defense by enhancing immunity [11]. Specific pathways, including pathogenesis-related proteins (PRs), phenylalanine ammonia-lyase (PAL) and reactive oxygen species (ROS) associated pathways, are active in the biochemical defense system, boosting plant defense by creating secondary bioactive metabolites [12].

To understand the compatible plant-pathogen interaction associated with the CMV infection of squash (*Cucurbita pepo* L.), the expression of different genes involved in plant defense pathways were studied in relatively early stages of infection (2, 4, 6, 8, 10, and 12 days after inoculation). Furthermore, an HPLC analysis of polyphenolic compounds was performed in the early stages of infection (6 and 12 days) in squash plants. We assayed levels of phenolic compounds as well as flavonoids in control and CMV-infected plants. Furthermore, we identified several chemical compounds accumulating in control vs. CMV-infected leaves using gas chromatography–mass spectrometric analysis (GC–MS).

## 2. Materials and Methods

### 2.1. Viral Isolate

Squash (*Cucurbita pepo* L.) plant samples exhibiting characteristics of CMV symptoms were gathered from open fields in the Alexandria governorate, Egypt. The collected samples were checked for viral infection using DAS-ELISA with a polyclonal antiserum against CMV (DSMZ, AS-0929) as previously described [13]. After checking positive ELISA samples by RT-PCR using specific primers as described previously, the CMV-infected squash leaves were ground using a mortar and pestle in 1:10 (*w*/*v*) of 0.1 M sodium phosphate buffer pH 7.0, containing 0.5% 2-mercaptoethanol. *Chenopodium amaranticolor* plants, as a local lesion host of CMV, were dusted with carborundum (600 mesh) and gently rubbed with a forefinger already soaked in the freshly prepared inoculum. Four to five days after mechanical inoculation of the virus, single local lesions that appeared on the leaves of *C. amaranticolor* were used as the origin of a pure viral isolate for squash inoculation and subsequent purification. 

### 2.2. Purification of CMV and Transmission Electron Microscopy 

The purified CMV isolate was prepared as previously described [14]. Briefly, 100 g of fresh systemically infected squash leaves were crushed in liquid nitrogen 12 days post-viral inoculation (dpi). The pulverized tissues were ground in 300 mL 0.5 M sodium citrate buffer (pH 6.5) with 0.2 mL thioglycolic acid and 200 mL chloroform. After 30 min of stirring, samples were centrifuged at 10,000 rpm for 10 min, the supernatant was added to 6% polyethylene glycol 6000 and 1% sodium chloride and stirred slowly overnight at 4 °C. After centrifugation at 10,000 rpm for 30 min, the pellets were resuspended in 80 mL of 0.01 M borate buffer and then centrifuged again at 10,000 rpm for 30 min to remove any non-soluble materials. The purified virus was scanned using a UV/VIS spectrophotometer Jenway 6405 (Cole-Parmer, Villepinte, France) to estimate the virus concentration using an extinction coefficient of 5 (mg/mL)^−1^ cm^−1^ at 260 nm. For transmission electron microscopy (TEM), formvar-coated nickel grids were floated on drops of purified virus preparations for 5 min. After a rinse with distilled water, the grids were stained with 2% phosphotungstic acid, pH 7.0, and viewed with transmission electron microscopy (TEM) (JEM-1400—Jeol Ltd., Tokyo, Japan). 

### 2.3. Molecular Characterization of CMV and Construction of a Phylogenetic Tree

According to the manufacturer’s protocol, viral RNA was extracted from purified virus solutions using a Plant Virus RNA Kit PVR050 (Geneaid Biotech 116 Ltd., New Taipei City, Taiwan). The cDNA synthesis was performed using M-MLV reverse transcriptase and oligo dT primers (Promega Corporation, Madison, WI, USA) with random hexamer primers in a 25 μL reaction mixture. RT-PCR was performed in a ProFlex™ PCR System (Thermo Fisher Scientific Inc., Applied Biosystems™, Waltham, USA) according to the manufacturer’s protocol. The synthesized cDNA was subjected to PCR using primers specific for the CMV coat protein gene and *Taq* polymerase (Promega, Madison, USA), according to manufacturer’s protocol [15] (Table 1). PCR conditions were the following: pre denaturation at 95 °C for 5 min, (95 °C for 1 min, 50 °C for 1 min, and 72 °C for 1 min) × 34 cycles, and the final extension step at 72 °C for 7 min. The PCR amplicons were electrophoresed in 1.5% agarose gels and TAE buffer, and visualized under a UV-transilluminator (Syngene, USA). A PCR clean-up kit (Maxim Biotech Inc., Rockville, MD, USA) was used to purify the amplified products according to manufacturer instructions, and purified amplicons were subjected to DNA sequencing. The obtained sequences were analyzed using the BLAST tool on the NCBI website to confirm sequence identity. Multiple sequence alignments were compared using MEGA 11 [16] to construct a phylogenetic tree.

### 2.4. Greenhouse Experimental Design and Sample Collection

Virus-free seeds of squash (*Cucurbita pepo* L.) cultivar Eskandarani were used during the current study. Seeds were purchased from the Agriculture Research Center, Egypt. Fifteen days after planting, the true top leaves of each squash plant were powdered with carborundum (600 mesh) and mechanically inoculated [17]. The experiment was performed in 10 pots, with three plants per pot. Six pots were CMV inoculated, and the rest served as controls. Plants inoculated with buffer only (mock) were used as controls. The collected squash leaves were from newer leaves, and the collection process was repeated every two days, on days 2, 4, 6, 8, 10, and 12 post-viral inoculation (dpi). All plants were maintained in an insect-proof greenhouse (28 °C day/16 °C night and 80% relative humidity), and 16-h daylight was used.

### 2.5. Determination of Defense Responses Using Real-Time Quantitative PCR (qPCR)

For all treatments, 100 mg of squash leaves were used as the starting material for total RNA extraction, accomplished using the guanidium isothiocyanate extraction method [18]. Using a Nano SPECTROstar spectrophotometer and gel electrophoresis, the quality of the RNA was determined. One µg of total RNA (pretreated with DNase I) from each sample was utilized as a template for cDNA synthesis, as reported previously [19]. Different primers were used to detect the expression of pathogenesis (PR) and polyphenolic-related genes listed in Table 1. Transcript (expression) levels were normalized to the housekeeping gene *EF1a* (Table 1). The RT-qPCR was performed in a reaction volume of 25 μL containing 1 μL of each primer (10 pmol/μL), 1 μL of template cDNA, 12.5 μL of 2X SYBR Master Mix (Fermentas, Waltham, MA, USA), and 9.5 μL of molecular biology grade water. Each sample was assayed in triplicate. The thermal program was performed in a QIAGEN rotor gene instrument (ABI System, Zanesville, OH, USA) with a program running at 95 °C for 10 min and 40 cycles (95 °C for 15 s, 60 °C for 30 s, and 72 °C for 30 s). A melting curve analysis was used to verify the PCR amplicons’ specificity. The relative expression ratio was accurately quantified and calculated according to the 2^-ΔΔCt^ algorithm [20].

### 2.6. Preparation of Ethanol Extract and HPLC Conditions

Squash leaves from all treatments were collected, air-dried, and pulverized. About 2 g leaves were extracted for 5 h in a shaking water bath at 40 °C with 15 mL of 99% ethanol. After filtration with Whatman no.1 filter paper, the filtered solution was moved to a fresh tube and condensed using a rotary evaporator. HPLC coupled with a Quad pump and a Zorbax Eclipse Plus C18 column (Agilent 1260 Infinity, Glendale, CA, USA) operating at 30 °C was used to detect the phenolic and flavonoid compounds, as previously reported [21].

### 2.7. GC–MS Analysis 

For GC–MS analyses, squash leaves from all treatments were extracted with ethyl acetate in a 1:1 (*v*/*v*) ratio for 20 min and then evaporated. Gas chromatography–mass spectrometry (GC–MS, TRACE 1300 Series, Thermo, Waltham, MA, USA) was used to analyze the leaf extracts’ secondary metabolites. GC–MS operation and conditions were applied as previously reported [22]. The compounds were recognized by matching them to GC–MS libraries and literature data.

### 2.8. Statistical Analysis

Using the CoStat program, the obtained data were statistically analyzed using a one-way analysis of variance (ANOVA) with *p* ≤ 0.05. Standard deviations (SD) of relative gene expression levels were used to display the significant differences. Relative transcriptional values over 1 (>1) indicate up-regulation, while values under 1 (<1) indicate down-regulation.

## 3. Results

### 3.1. Virus Isolation, Purification and Molecular Identification of the CMV-CP Gene

**Figure 1:**
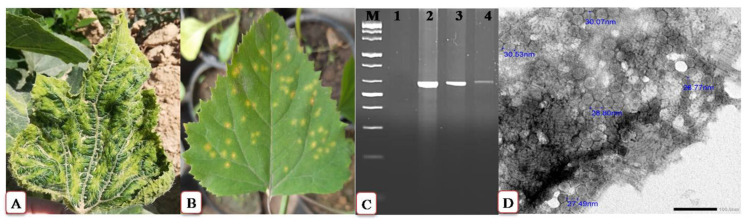
Squash (*Cucurbita pepo* L.) leaves showing mosaic-like symptoms, chlorosis, vein banding, and leaf malformation following a systemic infection by *Cucumber mosaic virus* (CMV) at 12 dpi (**A**). Single local lesions develop in CMV-inoculated *Chenopodium amaranticolor* leaves at 4–5 dpi (**B**). RT-PCR with primers specific for the *CMV-CP* gene detected an amplicon of 600 bp (M, 100bp ladder, 1, negative control, 2, CMV-infected squash, 3, and 4, *C. amaranticolor* local lesions induced by CMV) (**C**). Transmission electron microscopy (TEM) revealed that the purified CMV particles are spherical and approximately 30 nm in size (**D**).

**Figure 2:**
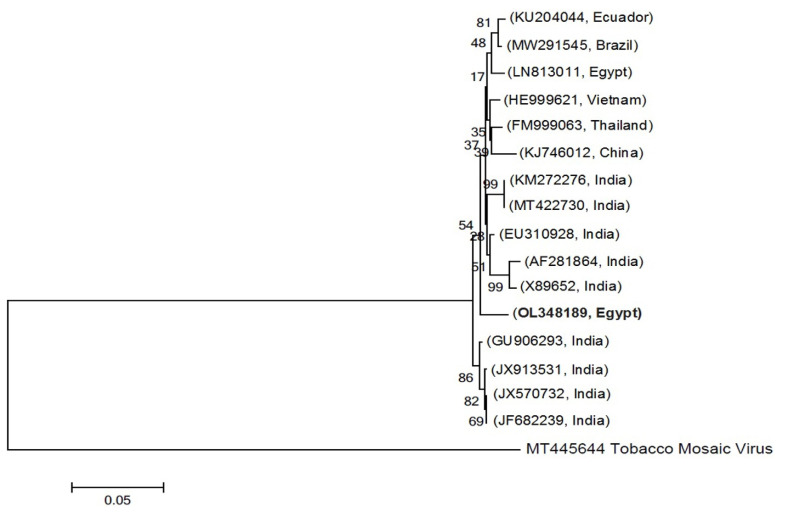
A constructed phylogenetic tree was drawn based on the sequences of the partial coat protein (CP) gene of an Egyptian *Cucumber mosaic virus* (CMV) isolate (OL348189) and other CMV isolates in GenBank.

The characteristic systemic symptoms of the field-collected squash samples were mosaic, chlorotic mottling, vein banding, blistering, and leaf malformation, as compared to healthy plants (Figure 1A). Approximately 95% of these samples tested positive for CMV in a DAS-ELISA test. Single local lesions developed in *Chenopodium amaranticolor* leaves (Figure 1B) 4–5 days post-viral inoculation (dpi) were used as a pure CMV isolate source for virus purification, molecular identification, and subsequent squash inoculation. The yield of the purified virus was about 4.6 mg/100 g of fresh weight of leaves. RT-PCR with primers specific for the *CMV-CP* gene detected a 600 bp-amplicon in the infected tissues (Figure 1C). A transmission electron microscope (TEM) study indicated that the purified CMV particles were spherical with approximately 30 nm in size (Figure 1D). Following PCR product purification and sequencing of the obtained amplicon three times, the annotated sequence was GenBank accessioned (accession number OL348189). The constructed phylogenetic tree showed that the Egyptian CMV isolate was closely related to two Indian isolates (Acc# AF281864 and Acc# X89652), with 99% similarity (Figure 2). 

Under greenhouse conditions, the squash plants mechanically inoculated with CMV started developing CMV-like symptoms at 10 dpi, with clearly visible characteristic symptoms at 12 dpi. The samples were collected every two days from 2 to 12 dpi. The collected samples of all treatments were subjected to DAS-ELISA as well as RNA extraction. The DAS-ELISA results revealed that CMV is detectable in inoculated squash plants at 6 dpi (ELISA value, EV of 0.391), as compared to the control (EV of 0.111). The CMV titer gradually increased, reaching the maximum ELISA value of 0.781 at 12 dpi.

### 3.2. Time-Course Expression of Defense-Related Genes

**Figure 3:**
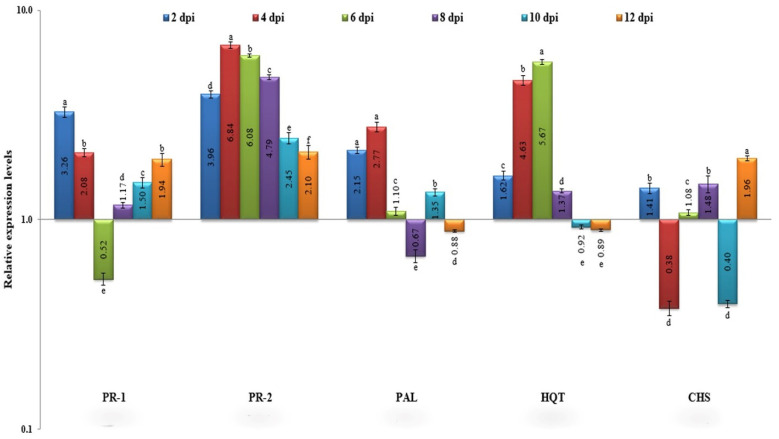
Relative expression levels of *PR-1*, *PR-2*, *PAL*, *HQT*, and *CHS* genes upon CMV infection from 2–12 dpi. The columns reflect the means of biological triplicates, while the bars represent the standard deviation (±SD). Small letters indicate changes between samples that are statistically significant. Values of columns with the same letter do not differ significantly.

The RNA extracts of all collected plant samples were used to synthesize cDNA and subsequently subjected to gene expression assays with RT-qPCR using different primers specific to defense-related genes. The RT-qPCR results of this study showed that *PR-1* was rapidly up-regulated once squash plants had been infected by CMV (Figure 3). The expression level was significantly greater (3.26-fold) than the mock at 2 dpi. Subsequently, the expression level decreased until it was 2.08-fold at 4 dpi and continued to decrease until it exhibited down-regulation with a relative expression level 0.52-fold lower than the control. This decrease may be related to the plant defense suppressing activity of CMV. At 8 dpi, the relative transcriptional values increased again, reaching an expression level 1.94-fold greater than the control at 12 dpi (Figure 3). The expression profile during the time-course study suggested that an early, transient induction of *PR-1* occurs in CMV–infected squash plants. Regarding *PR-2*, its expression exhibited a more dramatic response to viral infection as it was significantly up-regulated at all time points, as compared to the control (Figure 3). At 2 dpi, the gene was rapidly induced with a relative expression level 3.96-fold higher than that found in the mock treatment. The transcriptional level then increased to 6.84-fold at 4 dpi, 6.08-fold at 6 dpi, and 4.79-fold higher than the control at 8 dpi. The expression of *PR-2* then dramatically decreased at 10 dpi, reaching its lowest level at 12 dpi with an expression level only 2.10-fold higher than that of the control. The obtained results showed that CMV induced the expression of *PR-2* in the infected squash tissues at all of the time points studied (Figure 3).

Although CMV infection caused rapid induction of the *PAL* gene to 2.15-fold and 2.7-fold higher than the control at 2 and 4 dpi, respectively, the expression level then decreased dramatically, reaching the lowest level at 8 dpi with a relative transcription level 0.67-fold lower than the mock treatment (Figure 3). At 10 dpi, the expression level started to increase again reaching 1.35-fold and at 12 dpi the *PAL* gene transcriptional level was down-regulated by 0.88-fold compared to the control. For the transcriptional profile of *HQT* (encoding hydroxycinnamoyl CoA quinate transferase), the expression was initially slightly raised with a relative transcript 1.62-fold more than the mock plants at 2 dpi. The expression level of *HQT* then increased to 4.63-fold at 4 dpi, exhibited the highest transcriptional level (5.67-fold) at 6 dpi, and dramatically decreased to 1.37-fold at 8 dpi. At 10 dpi, a rapid down-regulation with a relative transcript level of 0.92-fold lower than the control was noticed. The CMV infection suppressed the expression of *HQT* at 12 dpi, where there was a relative transcript of 0.89-fold lower than control. The data shown in Figure 3 revealed that the transcription of *CHS* (encoding chalcone synthase) was induced at 2 dpi with a relative expression level 1.41-fold higher than that of the control. After that, *CHS* expression was completely shut down at 4 dpi and 10 dpi, with relative transcript levels of 0.38-and 0.40-fold lower than in the control. However, at 8 dpi, the relative transcript level was 1.48-fold higher than in the control (Figure 3), while at 12 dpi, *CHS* exhibited its highest transcriptional level of 1.96-fold greater than in the mock plants, implying that between 2 and 12 dpi, *CHS* transcript levels in CMV-infected plants fluctuated between up- and down-regulation.

### 3.3. HPLC Analysis of Phenolic Compounds in Ethanolic Squash Extracts

**Figure 4:**
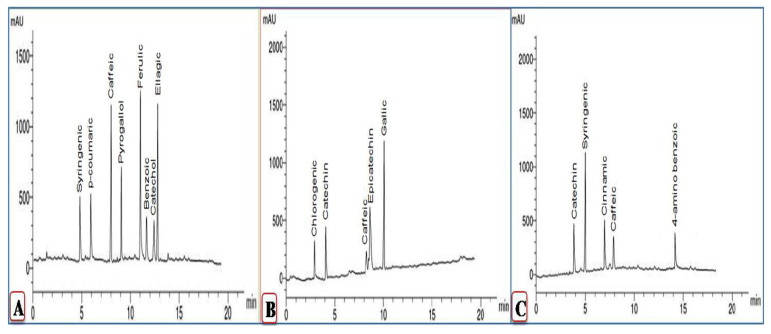
HPLC chromatograms of phenolic compounds in ethanolic extract of the squash plant. Mock-inoculated plants (**A**), CMV-inoculated plants at 6 dpi (**B**), and CMV-inoculated plants at 12 dpi (**C**).

**Figure 5:**
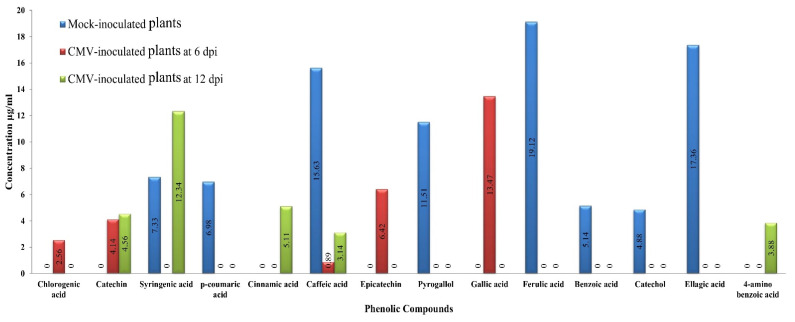
Comparison between the detected phenolic compounds and their concentration (µg/mL) in the ethanolic extract of the squash plants in mock-inoculated plants and CMV-inoculated plants at 6 and 12 dpi.

Figure 4 and Figure 5 present the phenolic compounds in ethanolic extracts of mock- and CMV-inoculated squash plants at 6 and 12 dpi. Compounds detected in the mock-inoculated plants were syringic, *p*-coumaric, caffeic, pyrogallol, ferulic, benzoic, catechol, and ellagic acid (Figure 5). In contrast, at 6 dpi, all of the 8 detected compounds except caffeic acid were entirely down-regulated upon CMV infection (Figure 4). By contrast, the accumulation of four phenolic compounds was detected in CMV-inoculated squash extracts (chlorogenic acid, catechin, epicatechin, and gallic acid), induced upon viral infection at 6 dpi. 

Three compounds, caffeic acid (15.63 μg/mL), ellagic acid (17.36 μg/mL), and ferulic acid (19.12 μg/mL), exhibited the highest levels in mock-treated squash extracts. In contrast, epicatechin (6.42 μg/mL) and gallic acid (13.47 μg/mL) showed the highest levels in CMV-infected extracts at 6 dpi. Similarly, most of the detected compounds in the mock-treated squash extract were down-regulated at 12 dpi. Out of the five compounds found in the CMV-inoculated squash extract at 6 dpi (chlorogenic acid, catechin, caffeic acid, epicatechin, and gallic acid), only two compounds, catechin (4.56 μg/mL) and caffeic acid (3.14 μg/mL), were induced upon CMV inoculation at 12 dpi. On the other hand, levels of two additional compounds, syringic acid (12.34 μg/mL) and cinnamic acid (5.11 μg/mL), were the highest in CMV-inoculated plant extracts at 12 dpi (Figure 4).

### 3.4. HPLC Analysis of Flavonoid Compounds in Ethanolic Squash Extracts

**Figure 6:**
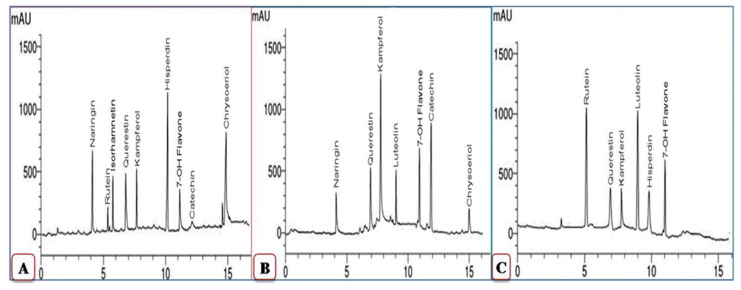
HPLC chromatograms of flavonoid compounds in ethanolic extract of the squash plant. Mock-inoculated plants (**A**), CMV- inoculated plants at 6 dpi (**B**), and CMV- inoculated plants at 12 dpi (**C**).

**Figure 7:**
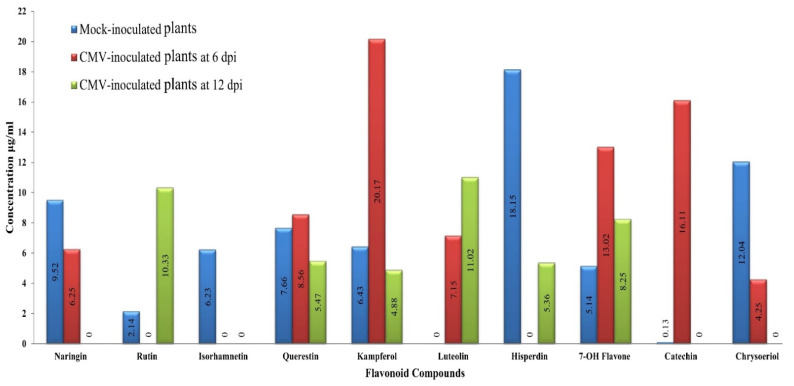
Comparison between the detected flavonoid compounds and their concentration (µg/mL) in the ethanolic extract of the squash plants in mock-inoculated plants and CMV-inoculated plants at 6 dpi, and 12 dpi.

Figure 6 and Figure 7 show the flavonoid compounds found in the ethanolic extracts of CMV-infected squash plants at 6 and 12 dpi for the two treatments. At 6 dpi, three compounds, rutin, isorhamnetin, and hisperdin, were down-regulated after CMV infection (Figure 6). On the other hand, an overaccumulation of three compounds, kampferol, 7-OH flavone, and catechin, with induction of luteolin were observed (Figure 6). The mock extract’s highest compound concentrations (9.52, 12.04, and 18.15 μg/mL) were displayed by naringin, chrysoeriol, and hisperdin, respectively (Figure 7). For CMV-infected extracts, three compounds, 7-OH flavone, catechin, and kampferol, exhibited the largest concentrations of 13.02, 16.11, and 20.17 μg/mL, respectively (Figure 7).

### 3.5. GC–MS Analysis of Ethanolic Squash Extracts

**Figure 8:**
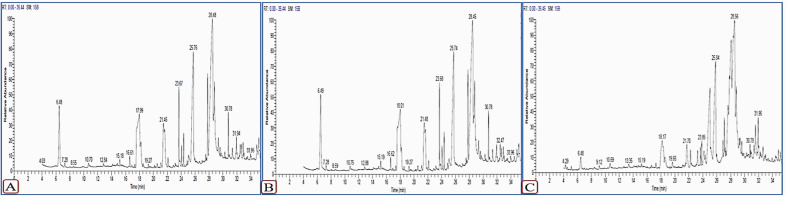
GC–MS chromatograms of ethanolic extract of squash plants. Mock-inoculated plants (**A**), CMV-inoculated plants at 6 dpi (**B**), and 12 dpi (**C**).

**Figure 9:**
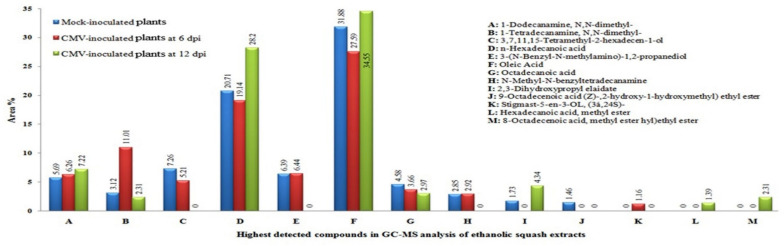
Comparison between the highest detected compounds and their area % (represented above the columns) in the ethanolic extract of squash plants in mock-inoculated plants and CMV-inoculated plants at 6 and 12 dpi.

Out of the 13 compounds with the highest concentrations detected in squash extracts, eight compounds, including 1-dodecanamine, *N*,*N*-dimethyl 1-tetradecanamine, *N*,*N*-dimethyl 3,7,11,15-tetramethyl-2-hexadecen-1-ol, n-hexadecanoic acid, 3-(N-Benzyl-N-methylamino)-1,2-propanediol, oleic acid, octadecanoic acid, and N-methyl-N-benzyltetradecanamine, were shared in the mock- and CMV-inoculated plants at 6 dpi with different concentrations (Figure 8 and Figure 9). On the other hand, 9-octadecenoic acid (Z)- and 2-hydroxy-1-(hydroxymethyl) ethyl ester were detected only in mock-inoculated squash extracts while stigmast-5-en-3-OL (3á,24S) was detected in CMV-inoculated squash plants extracts at 6 dpi only (Figure 8). The compound 2,3-dihydroxy propyl elaidate was present in the mock-inoculated plants but not in CMV-inoculated plants at 6 dpi, while it reappeared again at 12 dpi in increased concentrations. Two compounds, 3,7,11,15-tetramethyl-2-hexadecen-1-ol and 3-(N-benzyl-N-methylamino)-1,2-propanediol, were detected only in mock- and CMV-inoculated squash plants at 6 dpi. Meanwhile, the two compounds disappeared at 12 dpi. In contrast, hexadecanoic acid methyl ester, and 8-octadecenoic acid methyl ester ethyl ester compounds were observed in CMV-infected plants at 12 dpi only (Figure 9).

## 4. Discussion

*Cucumber mosaic virus* (CMV) has a wide distribution in Egypt [22]. In the current study, the CMV isolate (Acc# OL348189) identified and purified by us was also molecularly characterized based on CP gene sequence. This novel CMV isolate induced predictable symptoms, including severe chlorosis, yellowing, and mosaic symptoms, in mechanically inoculated squash plants. TEM and DAS-ELISA assays confirmed the identity of this virus isolate as CMV. These results are supported by the previous findings of Abdelkhalek et al. [22] and Farahat et al. [23]. In this regard, CMV titers assayed by ELISA were noticed to increase, especially and significantly at 6 and 12 dpi.

Phytochemical changes in squash plants upon CMV infection were investigated in this study. We wanted to elucidate the effects of exogenously applied CMV on defense responses of squash plants grown under greenhouse conditions. As a potential contribution to resistance to CMV invasion, squash tissues accumulate various phenolic compounds and induce defense-related genes, including those that encode pathogenesis-related proteins (PRs) [24,25]. In this regard, the RT-qPCR results of this study revealed that *PR-1* was rapidly induced in squash once CMV had infected the plants and down-regulated at 6 dpi, which may be related to the CMV activity that suppresses plant defense. The expression profile revealed in the time-course study suggested that an early, transient induction of *PR-1* occurs during the CMV–infection of squash. This result was similar to the findings of several studies that link the induction of *PR-1*, a systemic acquired resistance (SAR) regulator, to early defense responses [26,27,28]. Moreover, RT-PCR results of the *PR-2* gene showed increased expression levels upon CMV infection, as was shown for salicylic acid-inducible genes like *PR-2* and *PR-5* in systemic leaves of *Arabidopsis* plants infected by CMV [29]. Mayers et al. [30] found that SA induced resistance to CMV inhibited systemic virus movement in *Arabidopsis*, while in squash, it inhibited virus accumulation indirectly through inoculated tissues. Furthermore, the *PAL* gene is involved in SA and JA regulation in plants. Its expression kinetics confirm previous investigations that demonstrated viral infections to be associated with decreased *PAL* activity [21,31]. We found that, following CMV infection of squash, the expression of the *HQT* gene oscillated dramatically between the up- and down-regulated states. Polyphenolic molecules are produced primarily by the phenylpropanoid, flavonoid, and chlorogenic acid (CHA) pathways [32]. *HQT*, a crucial enzyme, increases CHA production in plant tissues [33]. As our RT-qPCR results revealed, CMV infection down-regulated the *HQT* gene at 10 and 12 dpi, which correlated with the disappearance of CHA at 12 dpi, as detected by HPLC. Meanwhile, different research groups have reported that CHA has a vital function in strengthening plant resistance to diseases [34,35,36]. Therefore, a successful infection by CMV may inhibit CHA synthesis in squash plants, leading to severe symptoms. CHS, the first essential enzyme in flavonoid biosynthesis in several plants, converts *p*-coumaroyl CoA to naringenin chalcones [37]. The down-regulation of CHS in CMV-infected tissues at 4 dpi suggests that the virus suppresses the synthesis of naringenin chalcones in the relatively early stages of pathogenesis. In fact, similar results were obtained by Abdelkhalek et al. [28] in TMV-infected tomato. 

Compared to mock plants, HPLC and GC–MS phytochemical analysis of squash plants during CMV infection at 6 and 12 dpi revealed the presence of many phenolic, flavonoid, fatty acid, methyl ester, and sterol compounds. Disease progression and stimulation of defensive mechanisms in infected plants rely heavily on bioactive molecules known as polyphenols [38]. Our data revealed that the three phenolic acids (chlorogenic acid, epicatechin, and gallic acid) appeared in CMV-infected plants only at 6 dpi. These results are consistent with those of Anuradha et al. [39], who found that the total phenolic content of *P. edulis* fruit was enhanced by *Telosma mosaic virus* infection. The phenolic compounds found in infected leaves and fruits were both up to 73% higher than in healthy leaves and 300% higher than in healthy fruits, according to studies by Jaiswal et al. [9] and Jabeen et al. [10]. On the other hand, according to Siddique et al. [40], if a plant membrane is disrupted during the pathogenic invasion, the plant host produces chlorogenic acid, which creates an adverse environment for the pathogen, and the resulting polyphenol oxides promote a further restriction of disease progress. In fact, the role of polyphenol oxidation in resistance to plant viruses like TMV has been known for a long time [41]. Meanwhile, Ashmawy et al. [42] proved the antimicrobial activity of chlorogenic acid against the bacterial isolates, *Pectobacterium carotovorum,* and *Dickeya solani*.

Elevated phenol levels in plants due to viral infections were also noticed by Rai et al. [43]. They concluded that higher than normal phenol levels enhance plant defense responses [40]. In fact, pathogen infection-induced increases in phenol levels may further accelerate phenol biosynthesis pathways. Once tomato plants were infected with CMV, the quantity of phenols in the plant increased, promoting the lignification of cell walls and contributing to plant resistance through an enhancement of the immune response [8]. According to the findings of several other studies, *Tomato yellow leaf curl virus* (TYLCV) infection also increased the amount of phenols in plants, as shown by Song et al. [44], El-Dougdoug et al. [45], Khalil et al. [46], and Jaiswal et al. [9]. According to Huston and Smith [47], tomato cultivars that have been infected with a virus have a higher concentration of phenolic compounds, which activate the plant’s defence mechanisms against the virus and increase the plant’s antioxidant capacity. However, although the number of phenolic compounds in the plant host was altered, the concentration of phenolic compounds was not affected by *Citrus tristeza virus* infection. Similarly, members of the *Passifloraceae* family were shown to display a 58.3% rise in the number of secondary metabolites, such as polyphenols and flavonoid compounds, following viral infection [48]. Our study shows that in CMV-infected squash, several phenolic compounds (*p*-coumaric, pyrogallol, ferulic acid, benzoic acid, catechol, and ellagic acid) disappear at 6 and 12 dpi, as compared to mock-inoculated plants, which suggests that CMV-infection may suppress phenolic acids that are produced in the highest amounts by infected plant cells. Our results regarding flavonoid accumulation in CMV-infected squash show that the up-regulation of certain compounds can be detected in an earlier stage of viral infection at 6 dpi, while other compounds accumulate at 12 dpi. Interestingly, however, three compounds (naringenin, isorhamnetin, and chrysoeriol) were down-regulated following CMV infection. In agreement with these results, CMV infection promoted the expression of flavonoid biosynthesis genes in *Luffa cylindrical* L. [49]. Also, TMV infection in tomato leaves induced significant modulation of *PAL* transcriptional levels as well as genes related to flavonoid production [50]. Gutha et al. [51] observed a considerable increase in flavonoid quantity in leaves infected with *Grapevine leafroll-associated virus-3*. These findings suggest that flavonoids may have a role in the physiological responses to viral infections. Sade et al. [52] reported similar findings, demonstrating that increased expression of *CHS* and *FLS* genes was correlated with increased tolerance to TYLCV infection.

Secondary metabolites are produced as part of a plant’s biochemical defense strategy, including compounds like sterols, fatty acids, and esters. The GC–MS analysis in the present study showed that the most prevalent secondary metabolites in leaf extracts of CMV-infected squash at 6 dpi were n-hexadecanoic and oleic acids, based on their peak areas. The concentrations of these two compounds further increased at 12 dpi. Although the antibacterial activities of n-hexadecanoic acid [53] and oleic acid [54] have been described, it seems that unsaturated or saturated fatty acids might not affect plant viral replication in plant cells. We hypothesize that the observed changes in phenolic and flavonoid accumulation in CMV-infected squash are caused by activation of the main phenylpropanoid pathway (as seen by elevated transcription of key genes), shifting plant defense processes to the formation of protective molecules such as flavonoids. Moreover, our results suggest that, at an earlier stage of CMV infection, phenolic spraying could potentially increase the resistance response in the squash host to control this viral disease. Our hypothesis is supported by the recent work of Akram et al. [55], who demonstrated that the foliar application of liquiritin, a plant flavonoid with a known role in promoting immune responses in humans and animals, can induce resistance to CMV in Chinese flowering cabbage plants under both greenhouse and field conditions. Importantly, the liquiritin-induced resistance to CMV is associated with enhanced levels of total plant phenolics. However, further investigations are needed for in-depth analyses of these viral pathogenesis processes.

## 5. Conclusions

In conclusion, this study suggests that CMV infection activates the phenylpropanoid pathway by inducing several genes that regulate phytochemical changes in squash plants. From 2 to 12 dpi, the transcript levels of *PR-1, PR-2, PAL, HQT*, and *CHS* genes show an early induction and subsequent suppression of the accumulation of phenolic and flavonoid compounds. HPLC and GC–MS analyses of CMV-infected squash extracts revealed that most of the phenolic and flavonoids compounds, fatty acids, and fatty acid methyl esters could be down-regulated upon viral infection. In a late stage of pathogenesis at 12 dpi, CMV suppresses the synthesis of phenolic acids (chlorogenic acid, *p*-coumaric acid, epicatechin, pyrogallol, gallic acid, ferulic acid, benzoic acid, catechol, and ellagic acid) and flavonoids (naringenin and isorhamnetin), leading to the virus’s rapid spread. We propose that the pathogen resistance of squash can be enhanced by foliar sprays of phenolics like chlorogenic acid at an earlier stage of CMV infection, to potentially control this viral disease. In summary, our data reveal new perspectives on squash stress tolerance, especially virus resistance, providing novel tools for further plant breeding strategies.

## Figures and Tables

**Table 1:** Primers used in this study.

Primer Name	Primer Code	Direction	Nucleotide Sequences (5′-3′)
Cucumber mosaic virus-coat protein	*CMV-CP*	Forward	GGATGCTTCTCCACGAG
		Reverse	AGTGACTTCAGGCAGT
Pathogenesis related protein-1	*PR-1*	Forward	CCAAGACTATCTTGCGGTTC
		Reverse	GAACCTAAGCCACGATACCA
Endoglucanase	*PR-2*	Forward	TCAATTATCAAAACTTGTTC
		Reverse	AACCGGTCTCGGATACAAC
Phenylalanine ammonia-lyase	*PAL*	Forward	ATGGAGGCAACTTCCAAGGA
		Reverse	CCATGGCAATCTCAGCACCT
Chalcone synthase	*CHS*	Forward	CACCGTGGAGGAGTATCGTAAGGC
		Reverse	TGATCAACACAGTTGGAAGGCG
Hydroxycinnamoyl CoA: quinate hydroxycinnamoyl transferase	*HQT*	Forward	CCCAATGGCTGGAAGATTAGCTA
		Reverse	CATGAATCACTTTCAGCCTCAACAA
Elongation factor 1-alpha	*EF1a*	Forward	ATTCGAGAAGGAAGCTGCTG
		Reverse	TTGGTGGTCTAAACTTCCAC

## Data Availability

Not applicable.
